# Vacancy-Engineered Nickel Ferrite Forming-Free Low-Voltage Resistive Switches for Neuromorphic Circuits

**DOI:** 10.1021/acsami.4c01501

**Published:** 2024-04-05

**Authors:** Rajesh Kumar R, Alexei Kalaboukhov, Yi-Chen Weng, K. N. Rathod, Ted Johansson, Andreas Lindblad, M. Venkata Kamalakar, Tapati Sarkar

**Affiliations:** † Division of Solid State Physics, Department of Materials Science and Engineering, Uppsala University, Uppsala SE-751 03, Sweden; ‡ Quantum Device Physics Laboratory, Department of Microtechnology and Nanoscience, Chalmers University of Technology, Göteborg SE-412 96, Sweden; § Division of X-ray Photon Science, Department of Physics and Astronomy, Uppsala University, Uppsala SE-751 20, Sweden; ∥ Division of Solid-State Electronics, Department of Electrical Engineering, Uppsala University, Uppsala SE-751 21, Sweden

**Keywords:** resistive switching, spinel ferrite, thin films, oxygen vacancy, long-term potentiation, short-term potentiation, pulsed laser deposition

## Abstract

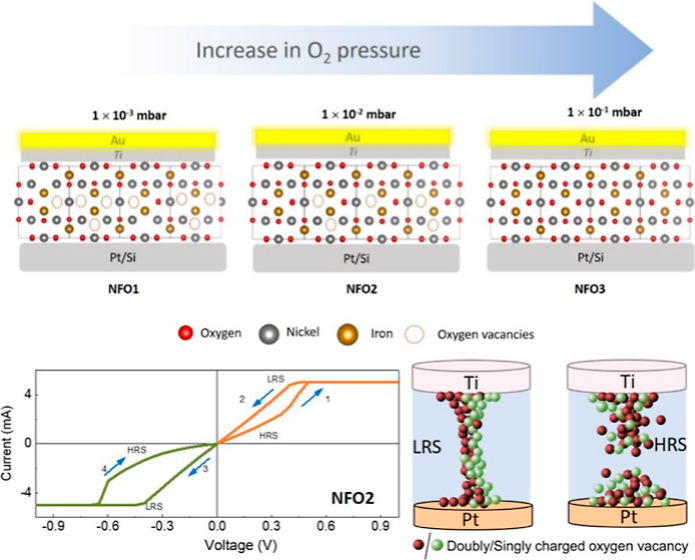

Innovations in resistive switching devices constitute a core objective for the development of ultralow-power computing devices. Forming-free resistive switching is a type of resistive switching that eliminates the need for an initial high voltage for the formation of conductive filaments and offers promising opportunities to overcome the limitations of traditional resistive switching devices. Here, we demonstrate mixed charge state oxygen vacancy-engineered electroforming-free resistive switching in NiFe_2_O_4_ (NFO) thin films, fabricated as asymmetric Ti/NFO/Pt heterostructures, for the first time. Using pulsed laser deposition in a controlled oxygen atmosphere, we tune the oxygen vacancies together with the cationic valence state in the nickel ferrite phase, with the latter directly affecting the charge state of the oxygen vacancies. The structural integrity and chemical composition of the films are confirmed by X-ray diffraction and hard X-ray photoelectron spectroscopy, respectively. Electrical transport studies reveal that resistive switching characteristics in the films can be significantly altered by tuning the amount and charge state of the oxygen vacancy concentration during the deposition of the films. The resistive switching mechanism is seen to depend upon the migration of both singly and doubly charged oxygen vacancies formed as a result of changes in the nickel valence state and the consequent formation/rupture of conducting filaments in the switching layer. This is supported by the existence of an optimum oxygen vacancy concentration for efficient low-voltage resistive switching, below or above which the switching process is inhibited. Along with the filamentary switching mechanism, the Ti top electrode also enhances the resistive switching performance due to interfacial effects. Time-resolved measurements on the devices display both long- and short-term potentiation in the optimized vacancy-engineered NFO resistive switches, ideal for solid-state synapses achieved in a single system. Our work on correlated oxide forming-free resistive switches holds significant potential for CMOS-compatible low-power, nonvolatile resistive memory and neuromorphic circuits.

## 1 Introduction

Research into next-generation memory storage has led to the development of novel nonvolatile memory elements,^1^ which are expected to propel the artificial intelligence revolution through neuromorphic computing applications^2,3^ and brain-inspired artificial learning.^4^ Unique among the different candidates for nonvolatile memory technology is resistance random access memory (RRAM),^5−8^ which relies upon switching a device between a high-resistance state (HRS) and a low-resistance state (LRS). An RRAM cell is fabricated in a simple industry-compatible capacitor-like structure in which an insulating oxide is sandwiched between two metal electrodes. RRAM exhibits key advantages, primary among which are its relatively simple structure [capacitor geometry-like metal–insulator–metal (MIM) configuration] and, hence, easy fabrication and better size scaling possibility, as well as good compatibility with the present-day complementary metal oxide semiconductor technology. Although RRAM devices that operate by the creation/dissolution of metallic filaments are becoming promising candidates for novel synapses and neurons,^9^ most of the studies in the MIM-based devices have difficulty in emulating both long-term potentiation (LTP) and short-term potentiation (STP) due to the stochastic nature of the relaxation process.^10,11^ In recent years, a variety of materials have been investigated as the switching layer including metal oxides like HfO_2_,^12^ TiO_2_,^13−15^ Ta_2_O_5_,^16^ and NiO^17^ as well as complex oxides belonging to the family of strongly correlated electron systems (SCES), e.g., BiFeO_3_^18^ and Pr_0.7_Ca_0.3_MnO_3_.^19^ In the SCES family, spinel ferrites^20^ are a technologically important and versatile member that have significance for nanoelectronics and spintronics.^21−24^ Here, highly insulating NiFe_2_O_4_ (NFO) has emerged as an important candidate. NFO belongs to the group of spinel ferrites^25^ that have the general formula AB_2_O_4_, with A and B referring to distinct crystallographic sites. While the A site is tetrahedral and surrounded by four oxygen ions, the B site is octahedral with six surrounding oxygen ions. Based on the distribution of divalent metal ions and trivalent Fe^3+^ ions on the A and B sites, the spinel ferrites can be either normal spinels (where the divalent metal cations occupy the tetrahedral A sites and the trivalent Fe^3+^ ions occupy the octahedral B sites) or inverse spinels (where the divalent metal cations occupy the octahedral B sites and the trivalent Fe^3+^ ions are shared equally between the octahedral and tetrahedral sites). NFO belongs to the inverse spinel group.

To explore the resistive switching capacity of NFO-based materials, elemental doping^26^ as well as embedding with nanoparticles and nanostructures^27,28^ have been explored. NFO thin films have been fabricated using different techniques such as reactive sputtering,^24^ spin coating,^26−32^ pulsed laser deposition (PLD),^33^ and sputtering.^34^ One key challenge that hinders the progress of RRAM technology is the inherent fluctuations during the write/erase operation, which originate from the stochastic nature of the formation and rupture of the conductive filament during the switching process, which is controlled by the oxygen vacancy concentration and their distribution and migration under electric fields. Another challenge here is also to reduce the electroforming voltage required for initial filament formation, which is often as high as 20–25 V.^27,28^ These high values of the electroforming voltage are caused by the need for a high electric field for vacancy creation.^35−37^ Engineering the oxygen stoichiometry can potentially allow a direct effect on the oxygen vacancy concentration in the switching layer for addressing these key challenges, and such innovation has not been performed in NFO. In particular, electroforming-free resistive switching, which allows for the creation of conductive filaments without an initial voltage, provides a promising approach to surmount the limitations of traditional resistive devices,^38−40^ which we target here.

**Figure 1:**
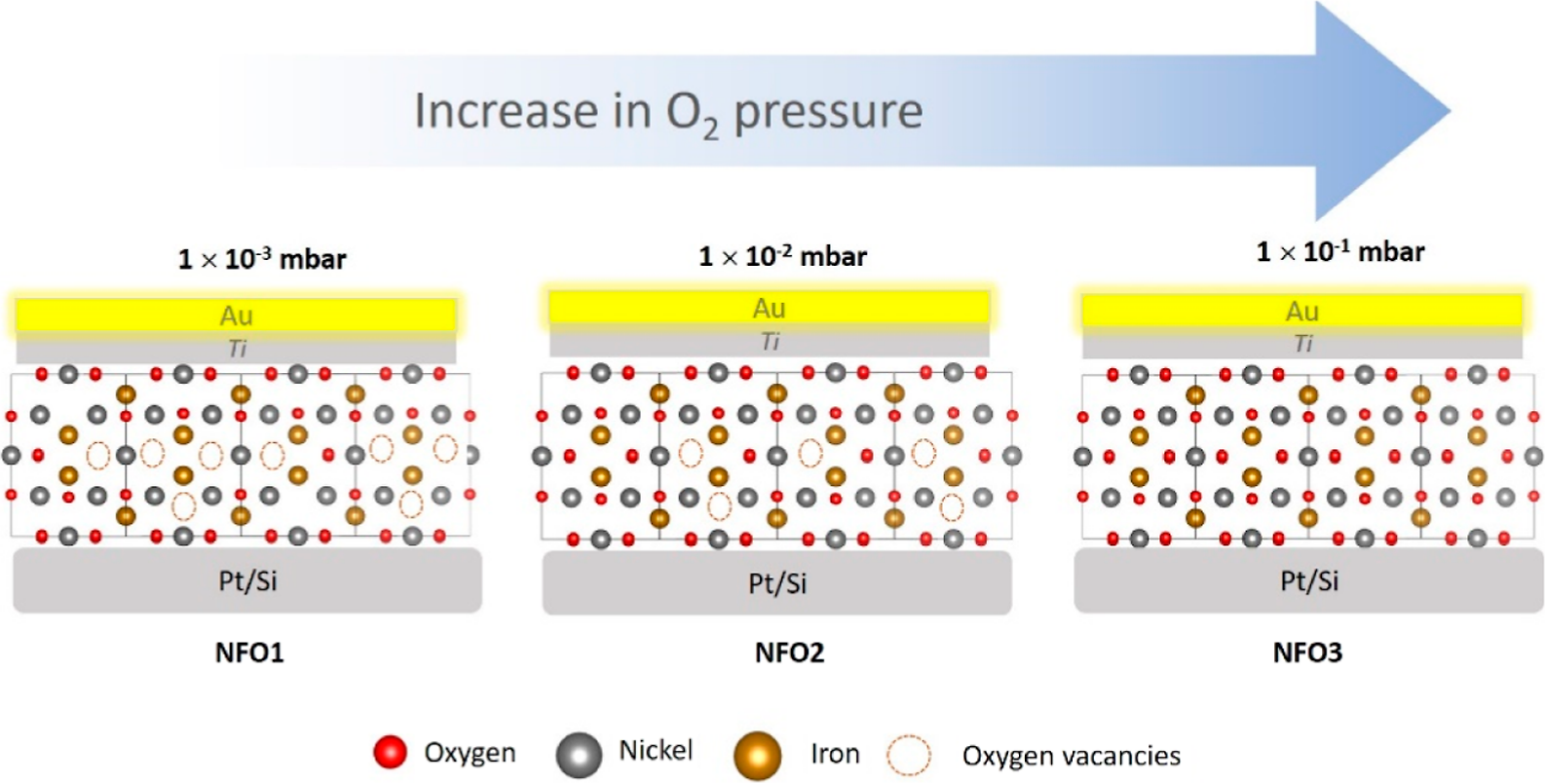
Schematic of the device structure and qualitative representation of the oxygen vacancies in the three films grown at different oxygen pressures and labeled as NFO1, NFO2, and NFO3.

In this work, we demonstrate the effect of the oxygen vacancy concentration on the resistive switching characteristics by varying the oxygen pressure during the fabrication process (Figure 1). Furthermore, by choice of the contact electrode, for the first time, we realize devices without applying any electroforming voltage that is necessary to initiate filament formation. This effect is studied in conjunction with titanium (Ti) as the top electrode (TE) to act as an oxygen reservoir that promotes a low power consumption forming-free resistive switching device. Strikingly, in addition to the concentration of oxygen vacancies, we also find a definite role of the cationic valence state and its effect on the charge state of the oxygen vacancies in the operation of the best-performing devices.

## 2 Experimental Section

### 2.1 Film Fabrication

The NFO films were fabricated by using PLD with a Lambda-Physik Compex 205 KrF excimer laser with a wavelength of 248 nm. Films with a thickness of ∼200 nm were fabricated on Pt-coated silicon substrates (MTI Corp., Richmond CA, USA) with a laser energy density on the target of 1.3 J/cm^2^ at 10 Hz repetition rate and a substrate temperature of 650 °C. The distance between target and substrate was set to 50 mm. The oxygen partial pressure was varied between 1 × 10^–3^ mbar to 1 × 10^–1^ mbar during the deposition. The flow of oxygen into the chamber was controlled by an electronic mass flow controller. The samples deposited at oxygen pressures of 1 × 10^–3^ mbar, 1 × 10^–2^ mbar, and 1 × 10^–1^ mbar are labeled as NFO1, NFO2, and NFO3, respectively. Before the deposition, the Pt-coated Si (Pt/Si) substrates were cleaned for 10 min in acetone and isopropyl alcohol in an ultrasonic bath.

### 2.2 Structural Characterization and Chemical Composition Information

Grazing incidence X-ray diffraction (GIXRD) was carried out by using a D5000 Siemens diffractometer. Raman spectroscopy was performed by using a Renishaw Raman spectrometer with a 532 nm laser beam. Before experiments, the Raman spectrometer was calibrated with a silicon reference to (520.5 ± 0.2) cm^–1^. In-house hard X-ray photoelectron spectroscopy (HAXPES) measurements were carried out with a Scienta Omicron EW4000 spectrometer, using a monochromatized Ga Kα radiation (*h*ν = 9.25 keV) source. The spectra were analyzed using the SPANCF package in Igor Pro 9.02.^41^ The spectra of Ni 2p, Fe 2p, and O 1s core levels were fitted in the least-squares sense with Voigt functions and a nonlinear Shirley background.

### 2.3 Electrical Characterization

Electrical characterization of the samples was performed using an Agilent B1500A semiconductor device parameter analyzer. Titanium (Ti)/gold (Au) contacts were deposited in a high-vacuum e-beam unit Lesker PVD 75 system by using a shadow mask. This resulted in rectangular electrodes of size 600 μm × 300 μm.

## 3 Results and Discussion

### 3.1 Structural Conformity of the As-Fabricated Films

**Figure 2:**
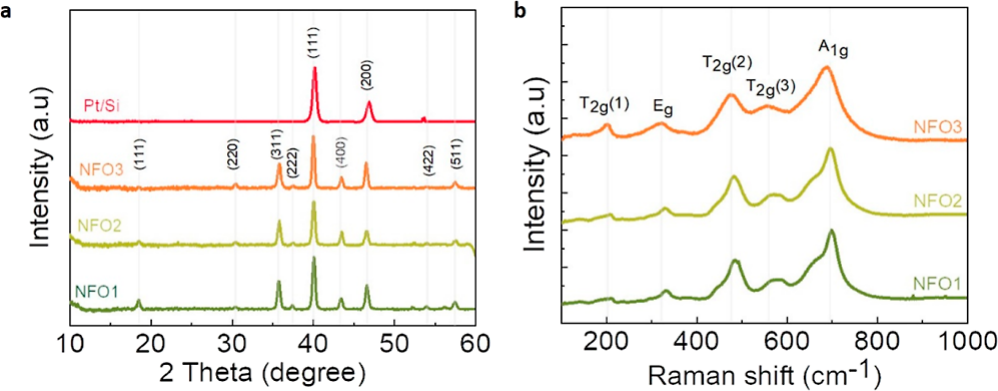
(a) GIXRD pattern of NFO thin films fabricated by PLD. (b) Raman spectra of NFO thin films on a Pt/Si substrate.

To confirm the structure of the films, the as-deposited NFO films were characterized by using GIXRD recorded by using a grazing incidence angle of 1°. The X-ray diffractogram in the 2θ region 10–60° for the three NFO films as well as a bare Pt/Si substrate is shown in Figure 2a. The Pt/Si substrate exhibits two high-intensity peaks originating from platinum, indexed as (111) and (200) (JCPDS card 04-0802). The NFO films show a polycrystalline nature without any secondary phase; all the peaks observed in the diffraction patterns could be indexed to the spinel phase of NFO (JCPDS card 54-0964).^32,33^

To obtain information about their internal structure, we further investigated the NFO films using Raman spectroscopy. The recorded Raman spectra of the films in the range of 100–1000 cm^–1^ are shown in Figure 2b. NFO exhibits five Raman active bands (A_1g_, E_g_, and three T_2g_ bands). The A_1g_ mode arises from the symmetrical stretching of oxygen atoms along the Fe–O or Ni–O bonds in the tetrahedral site. The *E*_g_ mode arises from the symmetrical bending of oxygen atoms with respect to the metal ion in the tetrahedral site. The T_2g_ (3) mode corresponds to the asymmetrical bending of oxygen,^42,43^ whereas the T_2g_ (2) mode corresponds to the asymmetric stretching of Ni–O and Fe–O bonds in the octahedral site.^44^ The translational motion of the metal ions along with the oxygen atoms in the tetrahedral coordination gives rise to the collective T_2g_ (1)^45^ mode. As can be seen in Figure 2b, all of the vibrational modes corresponding to NFO are present in our NFO films, further confirming the phase integrity of our samples.

### 3.2 Resistive Switching Characteristics

**Figure 3:**
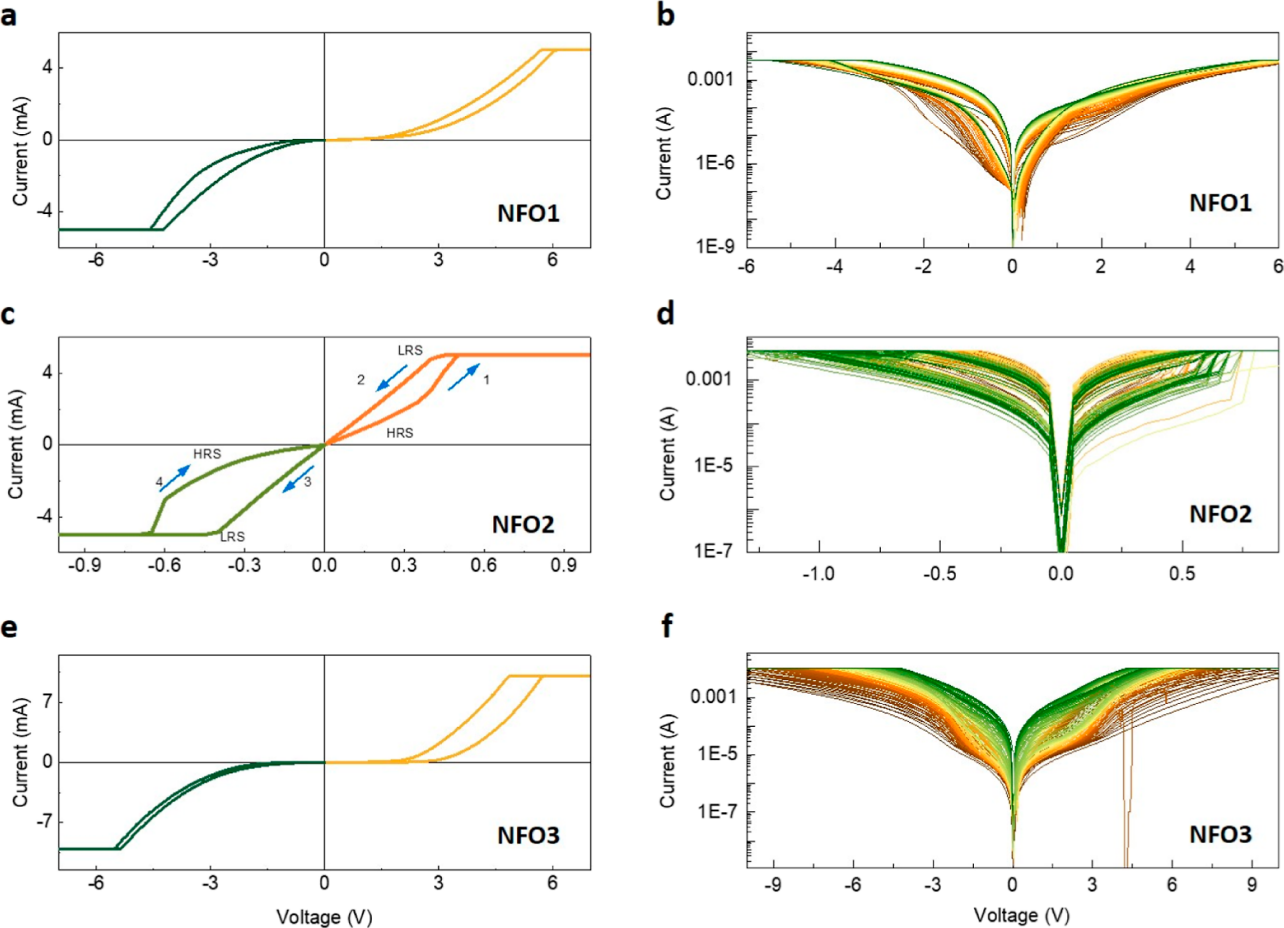
Room-temperature *I*–*V* curves of NFO samples. Left panel: First IV curve of (a) NFO1, (c) NFO2, and (e) NFO3. Right panel: 100 IV cycles measured continuously and plotted on a log scale for (b) NFO1, (d) NFO2, and (f) NFO3. The flat saturation is linked to the set current compliance.

Having confirmed the structural integrity of our films, we then characterized all three films electrically through room-temperature current–voltage (*I*–*V*) measurements to test their suitability for resistive switching applications. The *I*–*V* curves were measured continuously for 100 cycles to test at the same time the durability and stability of the films. In each cycle, first, the voltage was changed from 0 to +10 V, then back to 0 V (positive sweep), and then to −10 V and back to 0 V (negative sweep). No initial high voltage to trigger resistive switching (i.e., electroforming voltage) was applied. In Figure 3, we first present the obtained results for the three films and then discuss the implications.

In Figure 3, we show the *I*–*V* characteristics of the NFO samples. The first IV curves for the three films are shown in the left panel, and the corresponding curves on the right panel show 100 IV cycles for the corresponding films measured continuously. It is evident from these plots that NFO2 (NFO film deposited under an oxygen partial pressure of 1 × 10^–2^ mbar) shows clear low-voltage switching (<1 V) in a cycle between HRS and LRS, as expected for a typical memristor with bipolar resistive switching. It is worth noting that the transition from HRS to LRS occurring in the first cycle happens even before reaching the highest voltage of 1 V. NFO1 and NFO3, on the other hand, although displaying nonlinear IV curves and different resistive states, require higher voltages (closer to 10 V) for the switching. As shown in Figure 3d, even after 100 cycles, NFO2 shows persistent memristive behavior at low voltages, showing the robustness of the film.

**Figure 4:**
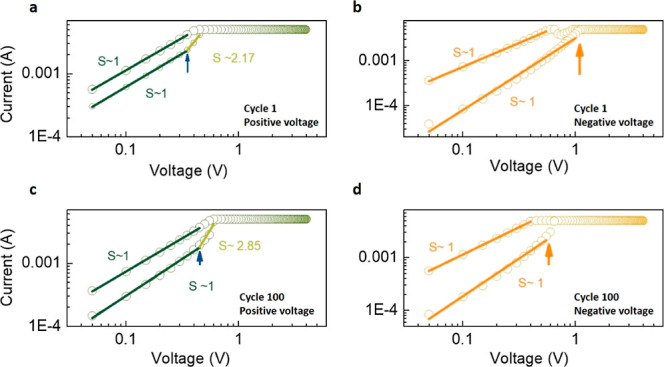
*I*–*V* curves of the NFO2 sample. Log–log plot of (a) cycle 1 in the positive voltage direction, (b) cycle 1 in the negative voltage direction, (c) cycle 100 in the positive voltage direction, and (d) cycle 100 in the negative voltage direction.

To understand further the actual transport mechanism in the best film (NFO2), and whether there is any change as we progress from the 1st cycle to the 100th cycle, we compared cycles 1 and 100 in more detail. In Figure 4, we show the log–log plots of current versus voltage for loop 1 (Figure 4a,b) and loop 100 (Figure 4c,d) of NFO2. The slope in the LRS is equal to 1, indicating Ohmic conduction in this region. In the HRS, we observe Ohmic conduction in the low-voltage region with the slope of the *I*–*V* curve equal to 1 up to a critical voltage (marked by arrows in Figure 4). Above this critical value, the slope increases to above 2, indicating a reduction in the potential barrier and increased flow of current, finally leading to switching to the LRS. Importantly, we do not observe any qualitative difference between cycles 1 and 100, indicating a robust and stable switching mechanism persisting over several cycles without any change. This is advantageous for device stability and endurance. A similar analysis of the IV curves of films NFO1 and NFO3 (Figure S1 and S2 in the Supporting Information, showing the IV log–log plots with space-charge-limited conduction^46,47^) shows instead a non-Ohmic behavior with many different slopes in different voltage regimes and different behavior of the device in cycle 1 compared to its behavior in cycle 100.

**Figure 5:**
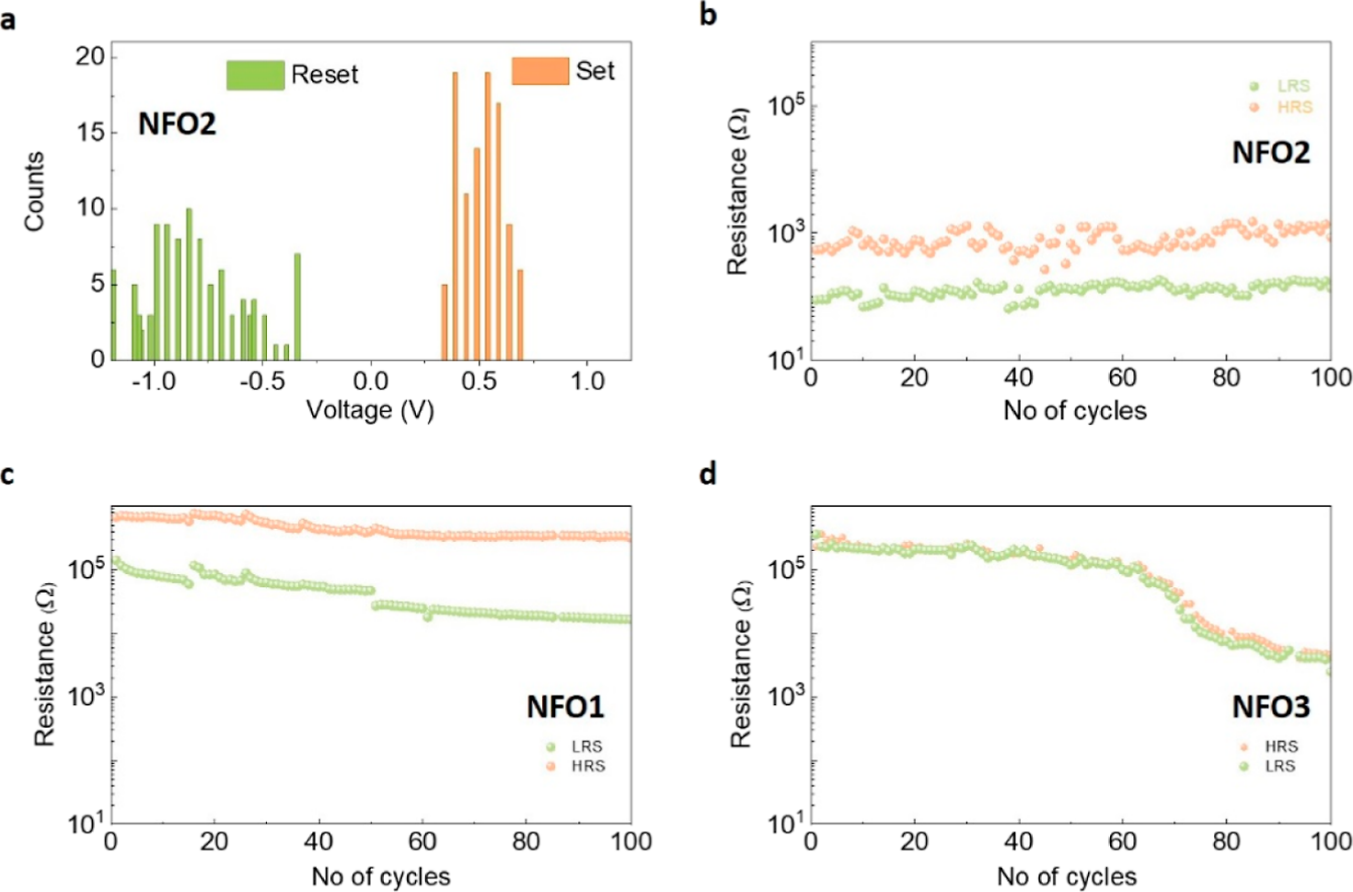
Endurance and stability of NFO samples. (a) Statistical distribution of SET and RESET voltages in NFO2 and calculated resistance values over 100 cycles at the readout voltage of 0.1 V for (b) NFO2, (c) NFO1, and (d) NFO3.

In Figure 5a, we show the statistical distribution of the SET and RESET voltages for NFO2, both occurring at relatively low voltages, with 100% of the SET processes occurring at voltages less than 1 V. The endurance of the film can also be observed in Figure 5b, which shows the resistance values in the LRS and HRS over 100 cycles at the readout voltage of 0.1 V. This clearly shows stable device performance without any degradation over 100 cycles. The LRS and HRS for NFO2 confirm a switching process with significant stability without any decay of resistance values over 100 cycles.

In contrast, in the absence of any electroforming process, NFO3 does not show the kind of robust and stable switching behavior observed in NFO2; in fact, it is not possible to distinguish the two resistive states in this film (Figure 5d). On the other hand, in NFO1, it is possible to distinguish between two distinct resistance states (Figure 5c), but only after the application of high electroforming voltages. Moreover, over 100 cycles, the resistance values in NFO1 start decaying, unlike the robust stability seen in NFO2. The decrease in resistance could be attributed to the creation of oxygen vacancies as a result of the electric field cycling.^48^ This decrease in resistance is also observed in NFO3 after ∼60 cycles, which could be attributed to field-induced vacancy creation; nevertheless, it never reaches a critical proportion so as to induce two distinct resistance states that is necessary for resistive switching.

**Figure 6:**
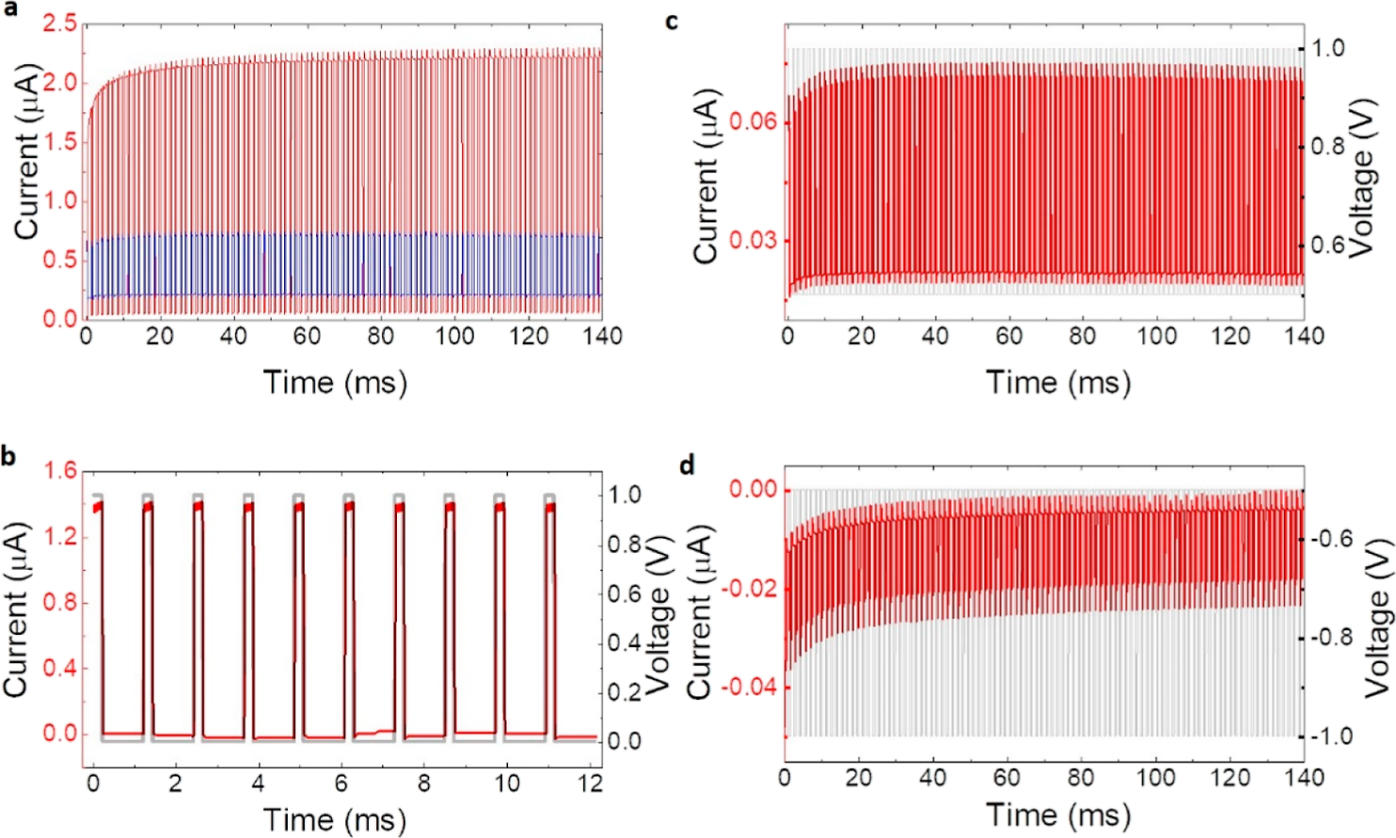
Transient response in the NFO2 sample. (a) Dynamic response of current vs voltage in the millisecond interval for 1 V (in blue) and 5 V (in red). (b) Current response for an applied voltage of 1 V for a longer waiting time interval. (c) Current response for positive 1 V in the millisecond time interval and (d) current response for negative 1 V in the millisecond time interval.

In addition to memory storage enabled by the two resistance states, long-term learning and short-term learning are two key features of neuromorphic memory. We selected NFO2, with its low-voltage forming-free switching and robust stability and endurance, as the ideal device to test the potential of the NFO resistive switches as synapses. Figure 6 illustrates the dynamic response of the NFO2 sample at various pulse intervals. We used voltage pulse widths of 0.2 ms. In Figure 6a, the current response to 1 (in blue) and 5 V (in red) pulses over millisecond intervals is displayed. The LTP, indicated by the film’s current response over time, is intensified when the voltage pulse is increased to 5 V, as evidenced by the heightened current spikes. To explore STP in the sample, the time interval was extended, as shown in Figure 6b. Here, the current response (in red) follows the voltage pulse amplitude and width (in gray), confirming STP behavior in the NFO2 sample. Further analysis of the dynamics in the NFO2 sample was conducted, with a focus on how it responds to fixed voltage pulse widths, aligned with LTP parameters. The effects of varying the direction and polarity of the voltage on the sample’s current response are demonstrated in Figure 6c,d. This is related to the capacity of the memristor to emulate synaptic functions such as potentiation and depression phenomena, which is crucial for the development of neuromorphic circuits. The potentiation and depression phenomena are related to the gradual change in resistance levels with the application of voltage pulses. Decreasing resistance (potentiation) and increasing resistance (depression) in an artificial synapse allow it to emulate the learning behavior of a biological synapse. This is seen in Figure 6c,d, respectively, where the application of short voltage pulses leads to a gradual decrease in resistance (Figure 6c). Once the memristor has reached its LRS, negative voltage pulses are applied (Figure 6d) and a gradual increase in the resistance is observed. These experiments reveal that shorter voltage pulse amplitudes result in both potentiation and depression effect, as desired.

### 3.3 Resistive Switching Mechanism

**Figure 7:**
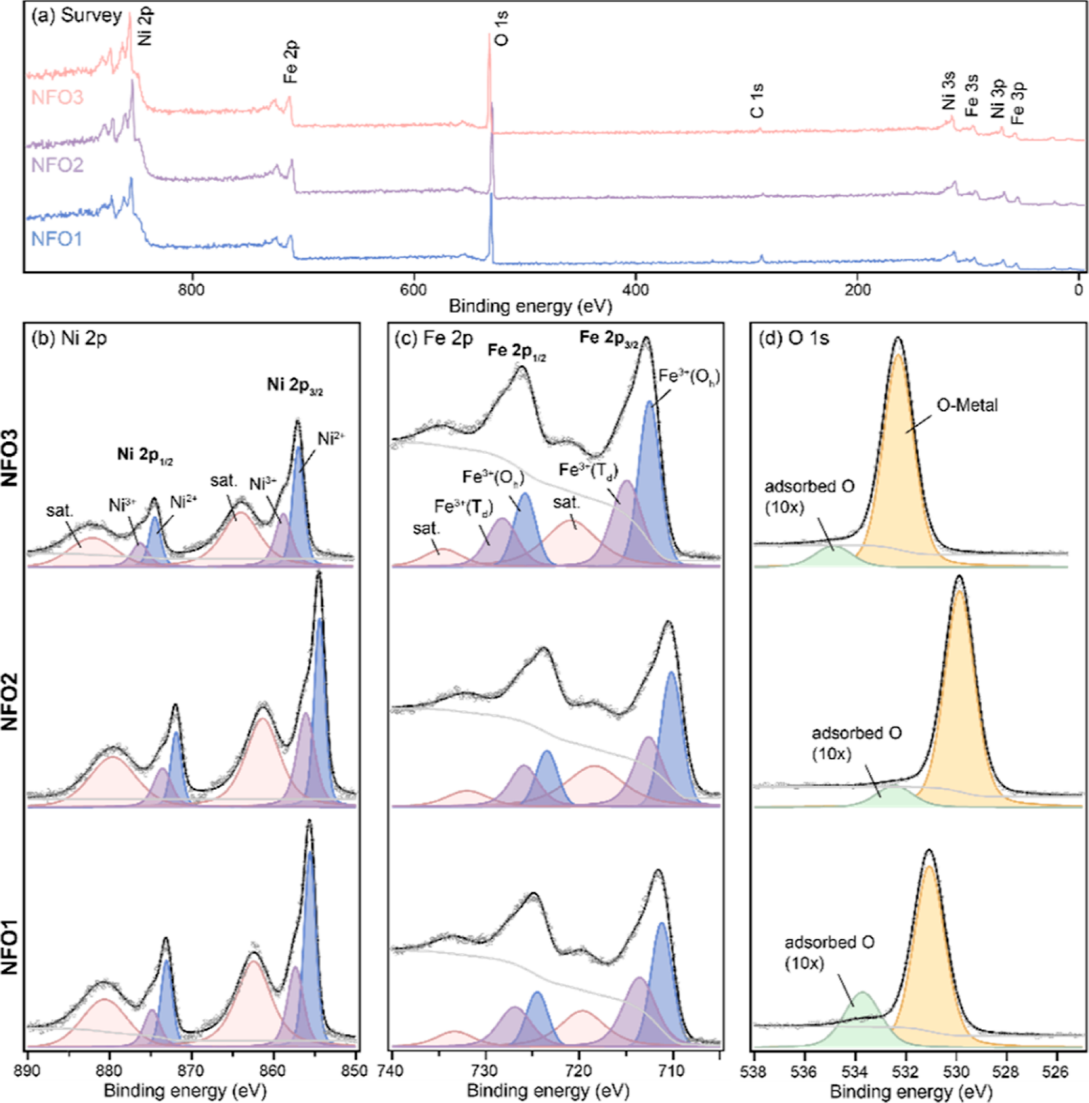
HAXPES on NFO films. (a) Survey scan, (b) Ni 2p, (c) Fe 2p, and (d) O 1s spectra of the NFO1, NFO2, and NFO3 samples. The satellites in the spectra are shortened as “sat”. Note that the peak corresponding to the adsorbed oxygen in the O 1s spectra is presented at 10 times the intensity of the original peak for better visibility.

Bipolar resistive switching primarily involves the migration of oxygen vacancies, which form and rupture conductive paths, altering the device’s resistance state. To clearly understand the mechanism of NFO resistive switching, we chose to first uncover the actual stoichiometry of our films using HAXPES. HAXPES analysis (*h*ν = 9.25 keV) was carried out on the NFO1, NFO2, and NFO3 samples to investigate their chemical state and composition. The inelastic mean free path, λ, for Ni at the photon energy of 9.25 keV with corresponding kinetic energy is about 7.95 nm.^49^ This gives an information depth of 23.85 nm (about 3 λ with 95% of the spectral information is obtained within the depth^50^), probing well beneath the surface region. The samples used for the HAXPES analysis were consistently sourced from those used in other characterization measurements. Figure 7a shows the survey scans of the samples. The C 1s peak has a higher intensity in the NFO1 sample. However, the overall intensity of C 1s for all samples is low, and there should be no carbon species in the sample; the source of the C 1s signal can be attributed to surface contamination from, e.g., CO_2_. The Ni 2p spectra in Figure 7b exhibit a spin–orbit splitting peak, Ni 2p_1/2_ and Ni 2p_3/2_, at the expected splitting energy of 17.5 eV.^51^ A further set of spectral features are identified as belonging to Ni^3+^ and Ni^2+^ states,^52^ suggesting nickel with mix-oxidation states occupying both octahedral and tetrahedral sites. The Ni^2+^/Ni^3+^ ratio (Table S1 in Supporting Information) was obtained using the intensity (area) of the fit functions, where NFO2 shows the lowest Ni^2+^/Ni^3+^ ratio. As shown in Figure 7c, the Fe 2p spectra show a spin–orbit split, Fe 2p_1/2_ and Fe 2p_3/2_, separated by 13.3 eV.^53^ The higher binding energy of the satellite is seen as an indicator for the presence of the Fe^3+^ state.^54−56^ The blue and purple peaks in the Fe 2p spectra were assigned to Fe^3+^ in octahedral (O_h_) and tetrahedral (T_d_) sites, respectively.^57,58^ For the ideal inverse spinel structure, the ratio of Fe^3+^ in the two lattice sites is equal to 1. We get a value very close to this for NFO1 and NFO2, while for NFO3, it deviates slightly (Table S1 in the Supporting Information). The O 1s spectra, as shown in Figure 7d, have a dominant signal between 529.8 and 532.3 eV that can be attributed to the metal–O bonding in the lattice.^53,58^ A minor peak in the spectra at a higher binding energy region is interpreted as the O-species adsorbed on the sample’s surface.^58,59^ The NFO1 sample shows the highest proportion of adsorbed O-species in O 1s, which supports the suggestion of the C 1s source from the surface contamination observed in the survey scan.

The HAXPES measurements described above reveal key features of the NFO films that are directly relevant to their resistive switching mechanism. We adjusted the oxygen vacancies in our NFO films by modifying the oxygen partial pressure during their PLD process. NFO1, fabricated at the lowest oxygen pressure, should have the maximum number of oxygen vacancies and should yield the best-performing resistive switching device. Contrary to this expectation, however, we observe that NFO2, fabricated at a moderate oxygen pressure, exhibits the most stable performance, with the resistance values in the LRS and HRS remaining unchanged over 100 cycles (Figure 5b), SET and RESET processes occurring at ∼1 V or lower (Figure 5a), much lower compared to that observed in the other two films, and the same IV curve in cycle 100 as in cycle 1 (Figure 4), indicating better endurance. To understand the superior performance of NFO2, we look more closely at the cation valences obtained from HAXPES (Table S1 in the Supporting Information). It is observed that out of the three films, NFO2 has the lowest Ni^2+^/Ni^3+^ ratio (∼30% lower than that of NFO1), indicating that nickel ions lose the maximum number of electrons in NFO2. These donated electrons can, in principle, be captured both by Fe^3+^ (to convert to Fe^2+^) and by the doubly charged oxygen vacancies (V_O_^2+^) to convert to singly charged oxygen vacancies (V_O_^1+^). However, the HAXPES results indicate a predominance of Fe^3+^ at the Fe site, suggesting that the majority of the electrons donated by nickel go to the oxygen vacancies. Thus, compared with NFO1, NFO2 has a lower (optimum) number of oxygen vacancies and a higher proportion of electrons donated by the nickel ions. These two factors combine to ensure that while NFO1 is largely populated by doubly charged oxygen vacancies (V_O_^2+^), in NFO2, the proportion of doubly charged oxygen vacancies (V_O_^2+^) and singly charged oxygen vacancies (V_O_^1+^) is more balanced.

The observed differences in the switching characteristics of NFO1 and NFO2 could be attributed to the differences in the concentration as well as the charge state of the oxygen vacancies. In NFO1, the presence of oxygen vacancies should, in principle, facilitate the electric-field-driven switching process via a field-driven drift of the positively charged oxygen vacancies. However, their very large number along with the fact that a majority of them are in the doubly charged state causes a strong repulsive electrostatic force to act between them when they form filaments, resulting in unstable filaments and stochastic behavior of the NFO1 device. A very high (above optimal) concentration of oxygen vacancies can also be detrimental to the formation of stable filaments due to the phenomenon of vacancy clustering. It has been observed previously^60^ that resistive switching timescales can be affected due to the differences in migration energy barriers of isolated and clustered oxygen vacancies. The differences in defect clusters and point vacancies in the context of bipolar resistive switching were also pointed out by Kim et al.^61^ A high degree of vacancy clustering can result in “frozen” oxygen vacancies. Therefore, creating devices with the optimal concentration of vacancies that can act as “free” defects is key for maximizing their efficiency for future memory and computing architectures.

Fewer stable filamentary conduction paths together with immobile oxygen vacancy clusters in NFO1 result in a higher overall resistance compared to that in NFO2 and switching at significantly higher voltages. In contrast, NFO2 has a lower (optimum) concentration of free and mobile oxygen vacancies, with many of them being converted from V_O_^2+^ to V_O_^1+^ by the uptake of electrons from the nickel sites. This will reduce the repulsive electrostatic force between them and lead to more stable filamentary conduction paths, resulting in a more consistent performance of the NFO2 device over several cycles, as we see from the measured IV curves (Figure 4) and the endurance and stability checks (Figure 5a,b). Interestingly, a recent theoretical study has observed that the singly charged oxygen vacancy state (V_O_^1+^) induces highly dispersed defect states where the carriers behave as free electrons leading to the formation of stable conducting filaments.^62^

**Figure 8:**
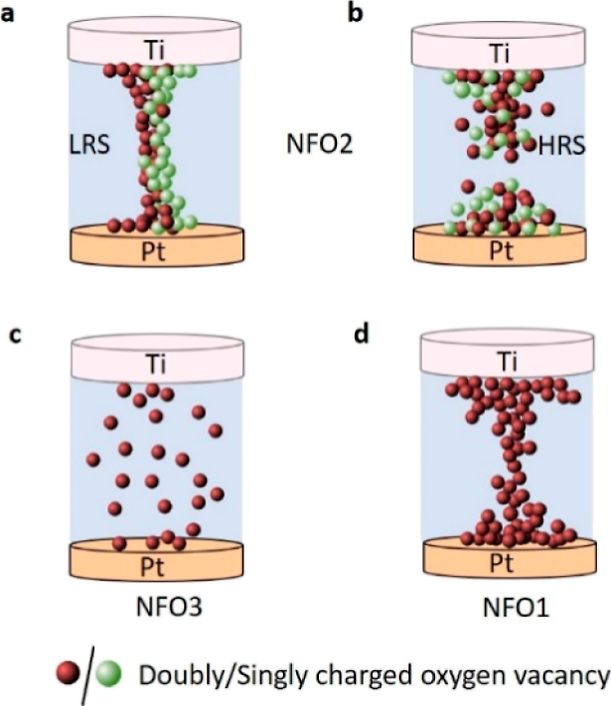
Schematic illustration of the switching mechanism in (a,b) NFO2, (c) NFO3, and (d) NFO1.

Thus, in NFO2, the adjustments in the cationic valency facilitate the creation of conduction paths at low voltages, a process known as SET, as depicted in Figure 8a. When the voltage polarity is reversed, these conductive filaments break, returning the device to its HRS, a mechanism known as the RESET process, as illustrated in Figure 8b. This leads to the manifestation of bipolar resistive switching in the device. Here, the inclusion of titanium (Ti) as the TE in the as-fabricated device plays a crucial role. It captures oxygen at the interface^63−65^ to create additional oxygen vacancies, which are essential for the device’s functionality. During the application of a positive voltage, some oxygen vacancies drift away from this interface. However, when a negative voltage is applied, these vacancies drift back. This dynamic process facilitates the formation and rupture of conductive filaments at low voltages. Consequently, the NFO2-based device becomes both forming free and low power consuming. We note here that all previous studies on understanding the resistive switching properties of NFO were done using symmetric electrodes of platinum (Pt) as both top and bottom electrodes.^26,31−33,66,67^

In the NFO3 sample, which was fabricated with a higher oxygen partial pressure, there are fewer oxygen vacancies in the initial film compared to what is required to create conduction paths, as shown in Figure 8c. Consequently, unlike NFO2, NFO3 lacks filamentary conduction paths. It does not show a switching between two distinct states.

The creation of oxygen vacancies under applied electric fields and their subsequent migration to enable resistive switching are especially important for asymmetrical devices,^68^ as in the present case. Spatially resolved studies like X-ray fluorescence maps have proved indicative of how oxygen vacancies are created and distributed on applying electric field in resistive switching memory devices.^69^ It was observed that the oxygen vacancies were introduced in the interface between the switching layer and the positively biased electrode.^69^ An interplay between the applied electric field and the number of oxygen vacancies in a device can also lead to a change in the switching mechanism.^68^ Below a critical electric field, if the number of oxygen vacancies is not sufficient to form stable conductive filaments (as in NFO3), or if they are “frozen” because of vacancy clustering (as in NFO1), they may instead act as trap centers, and the switching then operates via trapping and detrapping of carriers. On the other hand, if there are a larger (optimal) number of oxygen vacancies that are also free to migrate, the electric field then allows the formation and rupture of stable conduction filaments leading to ionic resistive switching (as in NFO2).

## 4 Conclusions

In summary, we have demonstrated optimal vacancy-engineered nickel ferrite forming-free low-voltage resistive switches by realizing asymmetric Ti/NFO/Pt heterostructures at room temperature. Through detailed structural, compositional, and electrical characterization of the as-fabricated films, we bring new insight into low-voltage forming-free resistive switching devices. Remarkably, our NiFe_2_O_4_ films clearly show both LTP and STP in the thin films with optimal vacancy concentration. X-ray photoelectron spectroscopy reveals a filament formation mechanism that can be tuned by carefully controlling the oxygen vacancies together with the presence of a Ni^2+^ deficiency in the samples. This promotes change in the charge state of the oxygen vacancies, leading to low-voltage forming-free resistive switching and robust, stable performance of the device. The presence of Ti as the TE also helps in inducing the formation of conduction paths with a moderate number of oxygen vacancies. The switching properties deteriorate as the number of oxygen vacancies deviates from the optimal number and in the absence of a Ni^2+^ deficiency. This study brings out the importance of controlling the oxygen stoichiometry as well as the charge state of the positively charged oxygen vacancies for developing low-power-consuming devices and CMOS-compatible neuromorphic circuits.

## Supporting Information Available

IV curves of NFO1 and NFO3 and intensity ratios obtained from HAXPES analysis (PDF)
