# Supplementary material for: Vacancy-Engineered Nickel Ferrite Forming-Free Low-Voltage Resistive Switches for Neuromorphic Circuits
Source: ACS Appl Mater Interfaces. 2024 Apr 5;16(15):19225–34. doi: 10.1021/acsami.4c01501 (PMC11040527; doi:10.1021/acsami.4c01501)
Supplement: Supplementary file 1 — am4c01501_si_001.pdf [file am4c01501_si_001.pdf]

Supporting Information for

Vacancy engineered nickel ferrite forming-free low-voltage resistive switches for neuromorphic circuits

Rajesh Kumar R [1], Alexei Kalaboukhov[2], Yi-Chen Weng[3], K. N. Rathod [1], Ted Johansson[4], Andreas Lindblad[3], M. Venkata Kamalakar[3‡], Tapati Sarkar[1*]

[1]Division of Solid State Physics, Department of Materials Science and Engineering, Uppsala University, SE-751 03, Sweden
[2]Quantum Device Physics Laboratory, Department of Microtechnology and Nanoscience, Chalmers University of Technology, SE-412 96, Göteborg, Sweden
[3]Division of X-ray Photon Science, Department of Physics and Astronomy, Uppsala University, SE-751 20, Sweden
[4]Division of Solid-State Electronics, Department of Electrical Engineering, Uppsala University, SE-751 21, Sweden

‡venkata.mutta@physics.uu.se
*tapati.sarkar@angstrom.uu.se

IV curves of NFO1 and NFO3

The log-log plots of current vs voltage of NFO1 and NFO3 show many different slopes in different voltage regimes, ranging from slightly above 1 to more than 4, as is seen in the presence of space-charge limited conduction[1–3]. The difference between the high resistance state and low resistance state is not clear i.e., the two branches overlap in many voltage regimes, unlike the clear difference observed for NFO2 (Fig. 4 in main manuscript). Furthermore, the robustness and stability exhibited by NFO2 is also missing, with the behavior of the device in cycle 1 differing substantially from its behavior in cycle 100. NFO1 also shows a cross-over point in the log-log plots of the I-V curves recorded in the positive voltage directions. However, the crossover point changes with respect to the number of cycles, indicating a stochastic and unpredictable behavior.

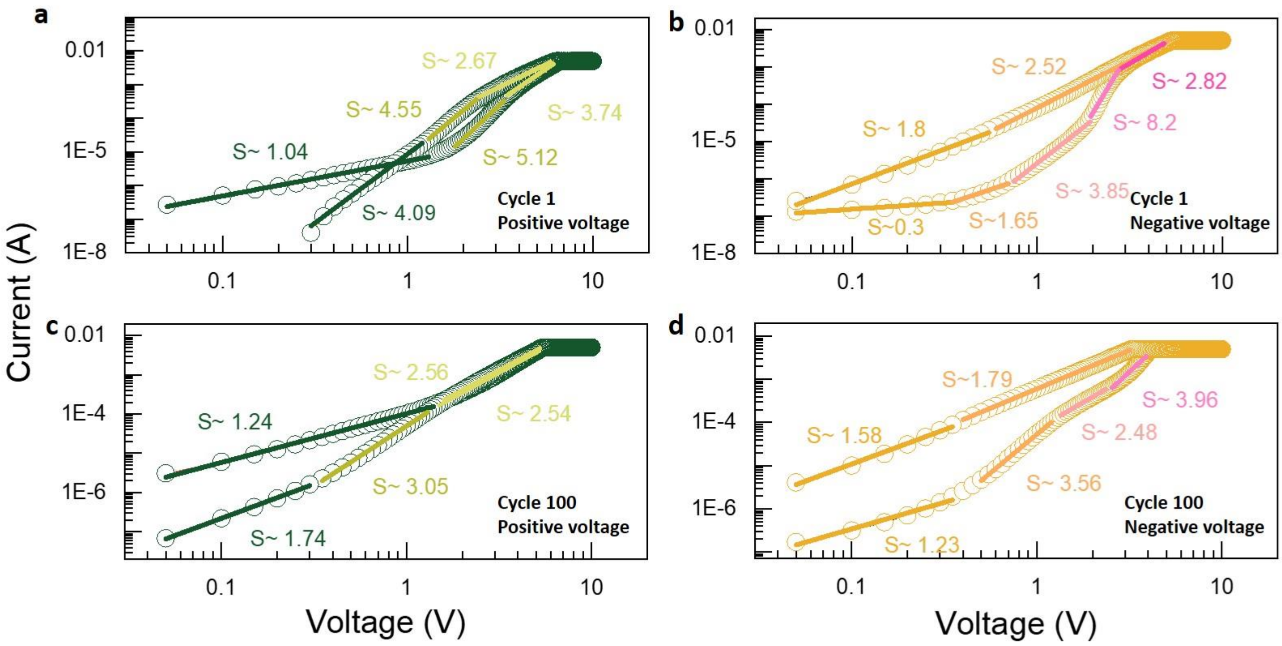

Figure S1. I-V curves of NFO1 sample. Log-log plot of (a) cycle 1 in the positive voltage direction, (b) cycle 1 in the negative voltage direction, (c) cycle 100 in the positive voltage direction, (d) cycle 100 in the negative voltage direction.

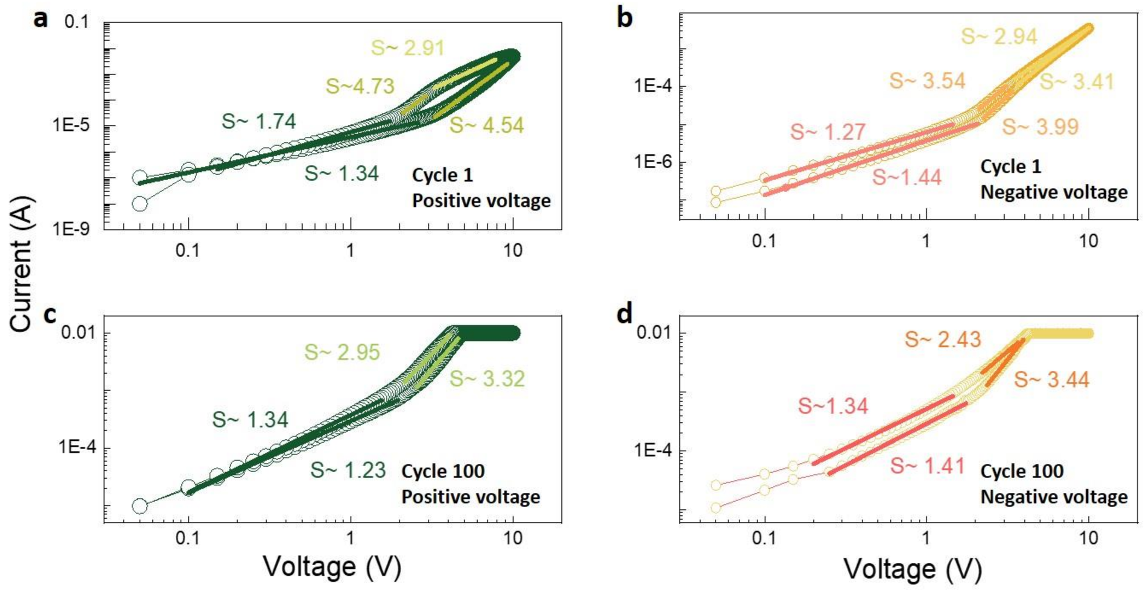

Figure S2. I-V curves of NFO3 sample. Log-log plot of (a) cycle 1 in the positive voltage direction, (b) cycle 1 in the negative voltage direction, (c) cycle 100 in the positive voltage direction, (d) cycle 100 in the negative voltage direction.

Intensity ratios from HAXPES analysis

	Ni^{2+}/Ni^{3+}	Fe^{3+}(Td)/Fe^{3+}(Oh)	Lattice O/adsorbed O
**NFO1**	1.64 ± 0.02	1.07 ± 0.01	27.9 ± 0.5
**NFO2**	1.13 ± 0.01	0.95 ± 0.01	81 ± 1
**NFO3**	1.41 ± 0.02	0.854 ± 0.004	84 ± 1

Table S1. Intensity ratios obtained from HAXPES data.

References

(1) Zhong, X.; Rungger, I.; Zapol, P.; Heinonen, O. Oxygen-Modulated Quantum Conductance for Ultrathin HfO_2-Based Memristive Switching Devices. *Phys Rev B* **2016**, *94* (16), 1–6. https://doi.org/10.1103/PhysRevB.94.165160.

(2) Ielmini, D.; Waser, R. *Resistive Switching: From Fundamentals of Nanoionic Redox Processes to Memristive Device Applications*; John Wiley & Sons, 2015.

(3) Kim, M. K.; Lee, J. S. Short-Term Plasticity and Long-Term Potentiation in Artificial Biosynapses with Diffusive Dynamics. *ACS Nano* **2018**, *12* (2), 1680–1687. https://doi.org/10.1021/ACSNANO.7B08331.
